# Genomic profiling of intestinal/mixed‐type superficial non‐ampullary duodenal epithelial tumors

**DOI:** 10.1002/jgh3.12632

**Published:** 2021-08-03

**Authors:** Shuichi Miyamoto, Goki Suda, Marin Ishikawa, Hideyuki Hayashi, Satoshi Nimura, Yoshihiro Matsuno, Ryo Mori, Shigeki Tanishima, Takahiko Kudo, Tomofumi Takagi, Yoshiya Yamamoto, Shoko Ono, Yuichi Shimizu, Naoya Sakamoto

**Affiliations:** ^1^ Department of Gastroenterology Hakodate Municipal Hospital Hakodate Japan; ^2^ Department of Gastroenterology and Hepatology Hokkaido University Graduate School of Medicine Sapporo Japan; ^3^ Genomics Unit, Keio Cancer Center Keio University School of Medicine Tokyo Japan; ^4^ Department of Pathology Fukuoka University Chikushi Hospital Fukuoka Japan; ^5^ Department of Surgical Pathology Hokkaido University Hospital Sapporo Japan; ^6^ Department of Biomedical Informatics Development Mitsubishi Space Software Co., Ltd. Tokyo Japan; ^7^ Department of Gastroenterology and Hepatology Health Science University of Hokkaido Sapporo Japan; ^8^ Department of Gastroenterology Japan Community Health Care Organization Sapporo Hokushin Hospital Sapporo Japan

**Keywords:** duodenal cancer, genomic testing, next‐generation sequencing, superficial non‐ampullary duodenal epithelial tumor

## Abstract

**Background and Aim:**

The mechanism underlying carcinogenesis and the genomic features of superficial non‐ampullary duodenal epithelial tumors (SNADETs) have not been elucidated in detail. In this study, we examined the genomic features of incipient SNADETs, such as small lesions resected via endoscopic treatment, using next‐generation sequencing (NGS).

**Methods:**

Twenty consecutive patients who underwent endoscopic treatment for SNADETs of less than 20 mm between January and December 2017 were enrolled. Targeted genomic sequencing was performed through NGS using a panel of 160 cancer‐related genes. Furthermore, the alteration/mutation frequencies in SNADETs were examined.

**Results:**

The maximum size of the SNADETs examined in this study was 12 mm in diameter. Five SNADETs were classified as low‐grade dysplasia (LGD) tumors, while 14 SNADETs were classified as high‐grade dysplasia tumors. Only one carcinoma in situ was detected. NGS data for 16 samples were obtained. APC alterations were detected in 81% of samples (13/16). KRAS, BRAF, and TP53 alterations were detected in 25% (4/16), 18.8% (3/16), and 6.3% (1/16) of cases, respectively.

**Conclusion:**

We detected *APC* alterations in most small SNADETs resected via endoscopic treatment, from LGD to carcinoma samples. Even in SNADETs classified as small LGD exhibited *KRAS* and *BRAF* alterations.

## Graphical abstract

*APC* alterations were detected in most small superficial non‐ampullary duodenal epithelial tumors (SNADETs) resected via endoscopic treatment. Even in SNADETs classified as small low‐grade dysplasia, *KRAS* and *BRAF* alterations were present in a few samples.

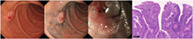

## Introduction

Superficial non‐ampullary duodenal epithelial tumors (SNADETs) are defined as adenomas and superficial adenocarcinomas, including carcinoma *in situ* (CIS) and submucosal invasive cancer of the non‐ampullary duodenal area.^1^ Duodenal epithelial tumors are extremely rare, with a reported prevalence of 0.4% in patients undergoing esophagogastroduodenoscopy.^2^ However, the detection rate of duodenal carcinoma has been increasing owing to the widespread use of endoscopy.^1^, ^3^

Recently, diagnostic methods based on magnified endoscopy with narrow‐band imaging (NBI) or endocytoscopy have been reported.^4^, ^5^ In addition, the number of resected SNADETs has been increasing owing to improvements in endoscopic treatment.^1^ Subsequently, our understanding of the clinical and pathological features of SNADETs has been improving.^6^, ^7^ However, relationships among the genomic profile and prognosis of SNADETs have not been clarified.

In colorectal cancer (CRC), the adenoma‐carcinoma sequence describes the process of carcinogenesis.^8^ *APC* plays a principal role in CRC development as a tumor suppressor gene. Extensive studies of associations between gene alterations in key driver genes and CRC metastasis^9^ have demonstrated the significant roles of alterations in *KRAS*, *TP53*, *SMAD4*, and *BRAF*. Similar mechanisms to those in CRC, such as the adenoma‐carcinoma sequence, may contribute to the pathogenesis of duodenal adenocarcinoma.^10^ Genomic analyses of duodenal tumors have reported *APC*, *KRAS*, and *BRAF* alterations.^11^, ^12^ Recently, numerous studies on genetic alterations of advanced small bowel adenocarcinomas have been reported.^13^ However, the data on SNADETs regarding genomic alterations are limited. In addition, the mechanism underlying carcinogenesis and the genomic features of SNADETs have not been elucidated in detail.

In this study, we examined the genomic features of incipient SNADETs, such as small lesions resected by endoscopic treatment, using next‐generation sequencing (NGS).

## Methods

### Subjects and samples

Twenty consecutive patients (20 samples) who underwent endoscopic treatment for SNADETs less than 20 mm in diameter between January and December 2017 at Hokkaido University Hospital were enrolled. Cold snare polypectomy (CSP) and endoscopic mucosal resection (EMR) are generally indicated for lesions that are ≤10 mm and ≤20 mm in diameter,^6^ respectively. Therefore, in this study, we included SNADETs that were less than 20 mm in diameter. None of the patients had any family history of cancer, familial adenomatous polyposis (FAP), or Peutz–Jeghers syndrome. SNADETs were removed by endoscopic treatment (EMR, CSP, or endoscopic submucosal dissection [ESD]).

This study was approved by the institutional review board of Hokkaido University Hospital (clinical research approval number 017–0417). Written informed consent was obtained from each participant. All experiments were performed in accordance with the ethical guidelines of the 2013 Declaration of Helsinki.

### Specimen handling

All resected specimens were routinely fixed in 10% buffered formalin for 24 h at room temperature. Thereafter, the specimens were serially sliced at a width of approximately 2 mm and embedded in paraffin following routine methods. All sections were cut to a thickness of 3 μm and stained with hematoxylin and eosin for microscopic examination. Paired peripheral blood samples were collected from each patient and stored at −80°C.

### Clinicopathological assessment

**Figure 1:**
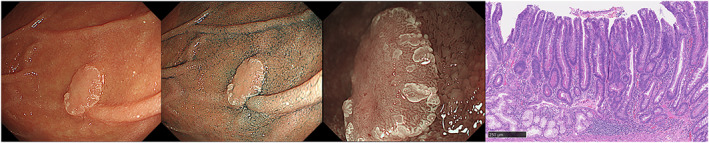
Low‐grade dysplasia (LGD) of superficial non‐ampullary duodenal epithelial tumors. Endoscopic and histopathologic images of LGD. (a) Endoscopic image with white light. The tumor was located in the second portion and detected as a slightly elevated lesion (10 mm in diameter). (b) Endoscopic image after spraying with indigo carmine. (c) Magnified endoscopic image with narrow‐band imaging. The surface pattern was preserved, and the vessel pattern was absent. (d) Resected LGD specimen composed predominantly of epithelial tubules. Nuclear polarity was well preserved. Paneth cells and goblet cells were recognized. (Hematoxylin and eosin, original magnification, ×130; scale bars, 250 μm).

**Figure 2:**
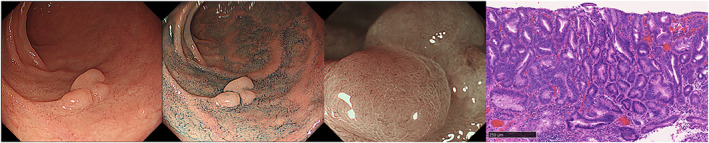
High‐grade dysplasia (HGD) of superficial non‐ampullary duodenal epithelial tumors. Endoscopic and histopathologic images of HGD. (a) Endoscopic image with white light. The tumor was located in the first portion and detected as a sessile‐type lesion (12 mm in diameter). (b) Endoscopic image after spraying with indigo carmine. (c) Magnified endoscopic image with narrow‐band imaging. The surface pattern was preserved, and the vessel pattern was like a network. (d) Resected specimen composed of various‐sized epithelial tubules, with focal loss of nuclear polarity, an increased nucleocytoplasmic ratio, and further loss of mucin production (hematoxylin and eosin, original magnification, 150×; scale bars, 250 μm).

**Figure 3:**
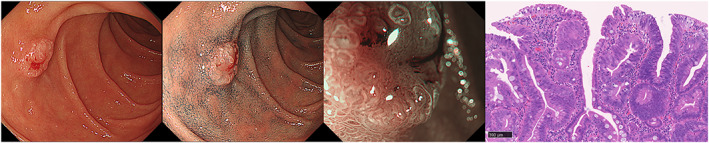
Carcinoma *in situ* (CIS) of superficial non‐ampullary duodenal epithelial tumors. Endoscopic and histopathologic images of CIS. (a) Endoscopic image with white light. The tumor was located in the first portion and formed as a slightly elevated and depressed lesion (8 mm in diameter). (b) Endoscopic image after spraying with indigo carmine. (c) Magnifying endoscopic image with narrow‐band imaging. The surface pattern was mixed (preserved and absent) and the vessel pattern was like a network. (d) Resected specimen composed of various‐sized epithelial tubules, showing a loss of nuclear polarity (hematoxylin and eosin, original magnification, ×180; scale bars, 100 μm).

Clinicopathological findings were reviewed, including age, sex, tumor location, tumor color, tumor size, tumor macroscopic type, resection method, histological type, and phenotype of the resected specimen. Macroscopic typing of SNADETs was based on the Japanese Classification of Colorectal, Appendiceal, and Anal Carcinoma.^14^ According to endoscopic features, the samples were classified into the elevated (0–I), superficial elevated (0–IIa), or superficial shallow or depressed types (0–IIc). Mixed patterns were diagnosed when more than one component was observed. Histological evaluations were performed by two expert pathologists (Satoshi Nimura and Yoshihiro Matsuno) who were blinded to the genomic analysis, clinical information, and endoscopic diagnosis. Histopathological diagnosis was based on the revised Vienna classification.^15^ Adenomas of the gastrointestinal tract can be categorized as low‐grade dysplasia (LGD; category 3) and high‐grade dysplasia (HGD; category 4.1). Adenomas were subclassified into low‐grade (equivalent to adenomas with mild to moderate atypia) and high‐grade (equivalent to adenomas with severe atypia) according to their degrees of structural and/or cytological atypia. CIS showed obvious structural atypia and nuclear atypia. Representative examples of these adenomas and CIS are shown in Figures 1, 2, and 3.

### Immunohistochemistry

Immunohistochemical staining was performed using the dextran polymer‐peroxidase‐based EnVision System (DAKO Japan, Tokyo, Japan) and metal‐3,3′‐diaminobenzidine (Pierce, Rockford, IL, USA). Finally, sections were counterstained with Mayer's hematoxylin. Membrane staining for CD10 (56C6; Novocastra, Newcastle, UK) and cytoplasmic staining for MUC2 (Ccp58; Novocastra) and MUC5AC (CLH2; Novocastra) were judged as positive when >5% of tumor cells showed a positive reaction for each marker. Based on CD10 expression and mucin phenotypes (MUC2 and MUC5AC) determined by immunoreactivity, SNADETs were further subclassified into five groups according to the criteria proposed by Yao *et al*.^16^: the small‐intestinal type was defined as CD10(+), MUC2(+/−), and MUC5AC(−); the large‐intestinal type was defined as CD10(−), MUC2(+), and MUC5AC(−); the gastric type was defined as CD10(−), MUC2(−), and MUC5AC(+); the mixed gastric and intestinal type was defined as MUC5AC(+), CD10(+/−), and MUC2(+); and the unclassified type was defined as CD10 (−), MUC2 (−), and MUC5AC (−).

### Genomic DNA extraction from tumor tissues and blood cells

Each resected specimen was sectioned into five slices (8‐μm‐thick slices), and macroscopic trimming was performed to obtain as many cancer cells as possible for more than 50% tumor cellularity. Genomic DNA was extracted from formalin‐fixed paraffin‐embedded (FFPE) tissue samples using a GeneRead DNA FFPE Kit (Qiagen, Hilden, Germany) according to the manufacturer's instructions. Genomic DNA was extracted from the blood samples using a genomic DNA extraction kit (Katayama Chemical, Osaka, Japan). The concentration and purity of genomic DNA samples were determined using a NanoDrop system (Life Technologies, Carlsbad, CA, USA) and Qubit dsDNA HS Assay Kit (Life Technologies) designed to be accurate for sample concentrations of 10–100 ng/mL. Genomic DNAs from the FFPE tissue and blood samples were stored at −80°C until analysis.

### Library construction and NGS

Multiplex PCR was performed using a GeneReadDNAseq Panel PCR Kit V2 (Qiagen) and Human Comprehensive Cancer Panel (Qiagen), which included 160 cancer‐related genes. Finally, an optimized library was constructed using a Gene Read DNA Library I Core Kit (Qiagen). The library was analyzed using an Agilent DNA 1000 Kit Bioanalyzer (Agilent Technologies, Santa Clara, CA, USA). Library preparation was achieved within two working days. The enriched libraries were sequenced to obtain paired‐end reads (2 × 150 bp) using the MiSeq NGS platform (Illumina, San Diego, CA, USA), resulting in a mean depth of >500×. The sequencing data were analyzed using an original bioinformatics pipeline, GenomeJack, tuned for clinical sequence examination, “CLUHRC” (Mitsubishi Space Software Co., Ltd., Tokyo, Japan).^17^

### Statistical methods

The results were analyzed using Prism version 6 (GraphPad Software, Inc., La Jolla, CA, USA). Data are expressed as means ± standard errors of the mean. Parameters were compared between two groups by Fisher's exact test or Student's *t*‐test. Differences were considered statistically significant when *P* < 0.05.

## Results

### Subjects and clinicopathological properties of the SNADETs

This study included 20 consecutive SNADETs resected by endoscopic treatment (Table 1). The maximum size of the tumors was 12 mm in diameter. The endoscopic procedures employed were CSP (11 lesions), EMR (8 lesions), and ESD (1 lesion). The case in which ESD was performed had severe submucosal fibrosis because of biopsy; therefore, we abandoned EMR and chose ESD for tumor resection. Most lesions (85%) were located in the second part of the duodenum. The phenotypic analysis showed no gastric‐type lesions. Intestinal‐type lesions were observed in 45% of cases (9/20 cases), and combined‐type lesions were observed in 55% of cases (11/20 cases). In this study, five SNADETs were LGD tumors (3 men, 2 women; mean age, 58.4 ± 3.37 years; mean diameter; 9.4 ± 1.17 mm; 0–I/0–IIa/0–IIc/0–IIa + IIc: 1/4/0/0). Moreover, 14 SNADETs were HGD tumors (10 men, 4 women; mean age, 63.0 ± 3.42 years; mean diameter, 7.14 ± 0.73 mm; 0–I/0–IIa/0–IIc/0–IIa + IIc: 1/7/4/2). Only one CIS in the SNADETs was detected (women; age, 83 years; mean diameter, 8 mm; 0–IIa + IIc).

**Table 1:** Clinicopathological characters of all 20 patients

Case	Age	Sex	Tumor location	Color	Resected method	Size (mm)	Macroscopic type	Phenotype	Histological type
2932	55	Male	Second portion	Red	ESD	6	0‐IIa	Intestinal type	HGD
0314	71	Male	Second portion	Red	CSP	8	0‐IIa	Combined type	HGD
5174	51	Female	First portion	Red	EMR	9	0‐IIa	Intestinal type	HGD
5824	67	Female	Second portion	Red	CSP	10	0‐IIa	Combined type	LCD
5768	51	Male	Second portion	Red	CSP	10	0‐lIa	Intestinal type	LGD
5455	79	Male	Second portion	Isochromatic	CSP	4	0‐IIa	Intestinal type	HGD
5490	71	Male	Second portion	Isochromatic	CSP	6	0‐IIa + IIc	Combined type	HGD
6697	66	Male	Second portion	Red	EMR	10	0‐I	Intestinal type	LGD
6944	55	Female	Second portion	White	CSP	5	0‐IIa	Intestinal type	LGD
7082	50	Female	Second portion	Red	EMR	5	0‐IIc	Combined type	HGD
6541	68	Male	Second portion	Red	EMR	10	0‐IIc	Intestinal type	HGD
7578	44	Male	Second portion	Red	EMR	5	0‐IIa	Combined type	HGD
7413	44	Male	Second portion	Red	EMR	4	0‐IIc	Combined type	HGD
7787	75	Male	First portion	White	CSP	12	0‐I	Combined type	HGD
7745	72	Female	Second portion	White	CSP	8	0‐IIa	Combined type	HGD
8290	81	Male	Second portion	Red	EMR	12	0‐IIa + IIc	Combined type	HGD
8454	83	Female	Second portion	Red	CSP	8	0‐IIa + IIc	Combined type	CIS
8654	66	Female	Second portion	Red	CSP	6	0‐IIa	Intestinal type	HGD
0131	53	Male	First portion	Red	EMR	12	0‐IIa	Combined type	LGD
8862	55	Male	Second portion	Isochromatic	CSP	5	0‐IIc	Intestinal type	HGD

CIS, carcinoma *in situ*; CSP, cold snare polypectomy; EMR, endoscopic mucosal resection; ESD, endoscopic submucosal dissection; HGD, high‐grade dysplasia; LGD, low‐grade dysplasia.

### Frequencies of gene alterations in SNADETs

**Figure 4:**
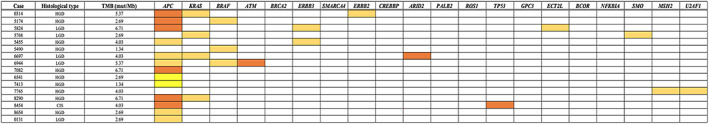
Gene alteration profiles of 16 tumors. Next‐generation sequencing results for 16 samples. There were no copy‐number variations. *APC* alterations were most frequent, followed by *KRAS*, *BRAF*, and *TP53* alterations. Additionally, alterations in *ATM*, *ERBB3*, *ARID2*, *ECT2L*, *SMO*, *MSH2*, and *U2AF1* were detected in some tumors. CIS, carcinoma *in situ*; HGD, high‐grade dysplasia; LGD, low‐grade dysplasia; SNV, single‐nucleotide variant; TMB, tumor mutational burden.

Twenty libraries were sequenced using NGS. Four libraries could not be analyzed owing to sample errors (low DNA yields or poor quality). Ultimately, we analyzed 16 libraries through NGS. There were no copy‐number variations. *APC* alterations were detected in 81% (95% confidence interval [CI], 54–96; 13/16) of cases. *KRAS*, *BRAF*, and *TP53* alterations were detected in 25% (95% CI, 7–52; 4/16), 18.8% (95% CI, 4–46; 3/16), and 6.3% (95% CI, 0.2–30; 1/16) of cases, respectively (Fig. 4). Alterations in *ATM*, *ERBB3*, *ARID2*, *ECT2L*, *SMO*, *MSH2*, and *U2AF1* were detected at low frequencies.

### Comparison of gene alteration profiles in LGD and HGD/CIS

**Figure 5:**
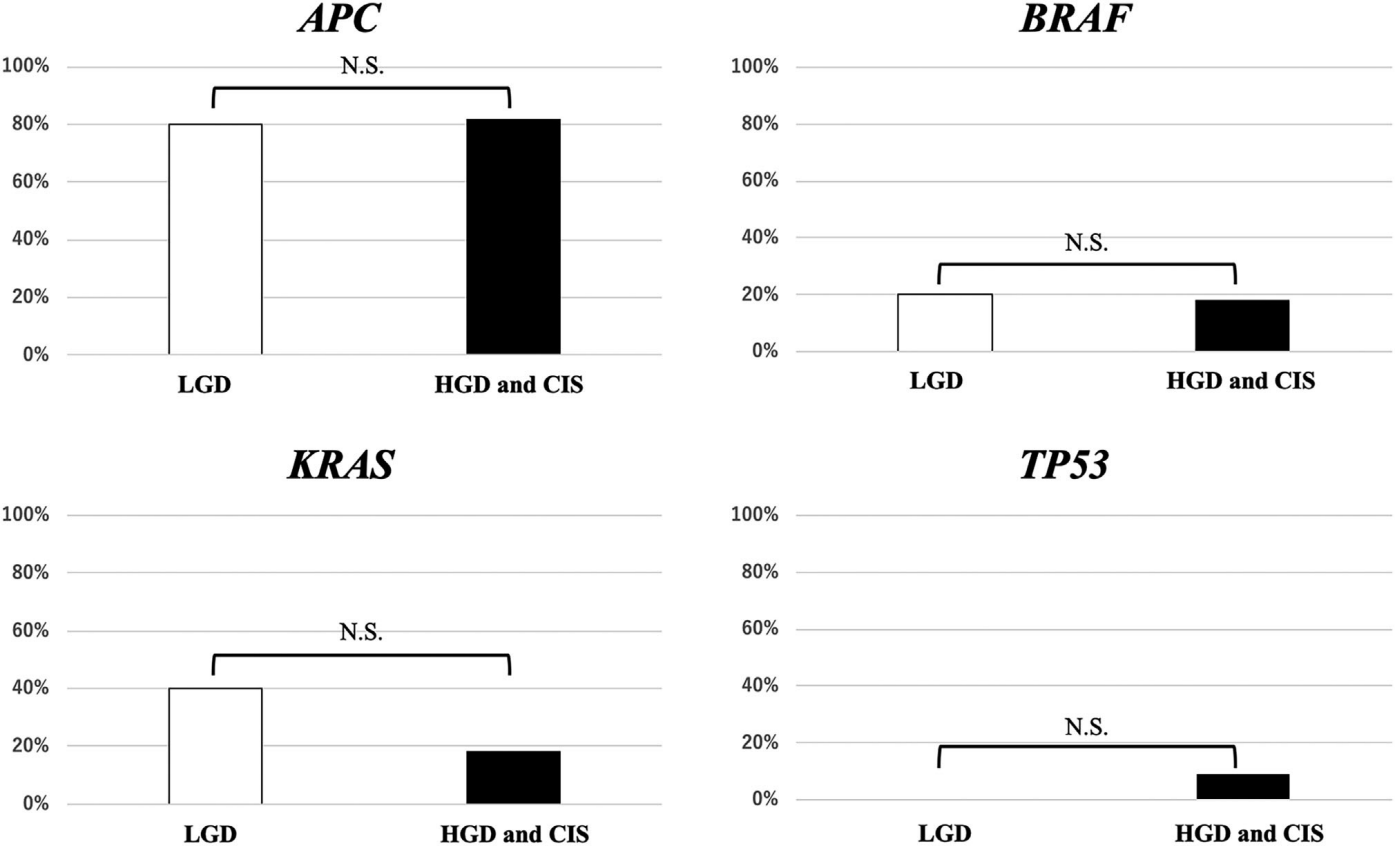
Comparison of gene alteration profiles between low‐grade dysplasia (LGD) and high‐grade dysplasia (HGD)/carcinoma *in situ* (CIS). The 16 samples analyzed using next‐generation sequence were divided into two groups (5 LGD and 11 HGD/CIS). There were no significant differences between the alteration frequencies of *APC*, *KRA*S, *BRAF*, and *TP53* in the LGD and HGD/CIS groups. Parameters were compared between two groups using Fisher's exact test. Differences were considered statistically significant if *P* < 0.05.

The 16 analyzed libraries were divided into two groups (5 LGD and 11 HGD/CIS). There were no significant differences between the rates of *APC* alterations in the LGD (4/5, 80%) and HGD/CIS groups (9/11, 81.9%), *KRAS* alterations in the LGD (2/5, 40%) and HGD/CIS groups (2/11, 18.2%), *BRAF* alterations in the LGD (1/5, 20%) and HGD/CIS groups (2/11, 18.2%), or *TP53* alterations in the LGD (0/5, 0%) and HGD/CIS groups (1/11, 9.1%) (Fig. 5). There were no significant differences between the alteration frequencies of any other genes in the two groups.

## Discussion

We observed a high frequency of *APC* alterations in SNADETs (i.e. 81%). Additionally, there were no significant differences between the rates of *APC* alterations in the LGD group (80%) and the HGD/CIS group (81.9%). Kojima *et al*. reported an *APC* alteration frequency of 54.5% in duodenal adenoma.^12^ *APC* plays a critical role in CRC development as a tumor suppressor gene, and its gene product inhibits Wnt/β‐catenin signaling.^18^ Based on a gene set enrichment analysis, Sakaguchi *et al*.^11^ found a strong association between expression profiles in duodenal adenomas/adenocarcinomas and colorectal adenomas after Cre‐lox *APC* knockout. These findings suggest that upregulation of the Wnt/β‐catenin pathway is a major factor in the initial stages of duodenal adenoma/adenocarcinoma carcinogenesis. Our results further support the key role of APC in duodenal adenomas/adenocarcinomas.

In CRC, *BRAF* and *KRAS* alterations typically arise at the adenoma stage of the adenoma‐carcinoma sequence,^19^, ^20^ following an initial *APC* alteration. *KRAS* and *BRAF* encode proteins belong to the Ras–Raf–MEK–ERK signaling pathway. The activation of this pathway is considered a molecular switch, leading to cell growth and proliferation.^21^ Alterations in *KRAS* and *BRAF* are associated with a risk of developing advanced neoplasia^22^ and contribute substantially to CRC metastasis.^9^ In the present study, *KRAS*, *BRAF*, and *TP53* alterations were detected in 25, 18.8, and 6.3% of patients, respectively. Surprisingly, we detected *KRAS* and *BRAF* alterations in 40% (2/5) and 20% (1/5) of LGD lesions, respectively. These findings are consistent with a previous study showing that one in five cases of LGD (20%) harbor a *KRAS* alteration.^12^ It has been reported that even in cases of LGD, large SNADETs of ≥20 mm in diameter exhibit a high risk of progression to adenocarcinoma.^23^ There were no histological differences between LGD tumors with *KRAS* or *BRAF* alterations and those without alterations within wild‐type sequences.

*TP*53 is a key driver gene in CRC progression and is frequently detected in small bowel advanced adenocarcinoma.^13^ In this study, one case of CIS had a *TP*53 alteration. These results support the hypothesis that the accumulation of genetic alterations after an initial APC might cause progression from adenoma to carcinoma in SNADETs. Considering our results and those of previous reports,^11^ SNADET progresses according to an adenoma‐carcinoma sequence, similar to colorectal tumors. Additionally, more than half of the LGD SNADETs (60%; 3/5) already had *KRAS* or *BRAF* alterations, which might result in progression to HGD or carcinoma.

This study had several limitations. It included a relatively limited number of samples (20 samples) and did not include submucosal invasive cancer samples. There were no lesions with a gastric phenotype in the collected samples. Additionally, we performed genome sequencing analysis using the Human Comprehensive Cancer Panel (Qiagen), which included 160 cancer‐related genes. Therefore, we could not analyze other gene alterations and epigenomic changes in SNADETs. These limitations should be considered when interpreting the study results. Therefore, further studies that include a larger number of cases and lesions with a gastric phenotype are needed in the near future.

In conclusion, in the incipient SNADETs, such as small lesions resected by endoscopic treatment, we detected *APC* alterations in most SNADETs from LGD to carcinoma samples. Even in SNADETs classified as small LGD (<12 mm in diameter), *KRAS* and *BRAF* alterations were present in few samples.
